# Perceived determinants of physical activity among women with prior severe preeclampsia: a qualitative assessment

**DOI:** 10.1186/s12905-022-01692-3

**Published:** 2022-04-27

**Authors:** Lili L. Kókai, Marte F. van der Bijl, Martin S. Hagger, Diarmaid T. Ó Ceallaigh, Kirsten I. M. Rohde, Hans van Kippersluis, Jeanine E. Roeters van Lennep, Anne I. Wijtzes

**Affiliations:** 1 Department of Public Health, Erasmus MC, Rotterdam, The Netherlands; 2 Department of Psychological Sciences and Health Sciences Research Institute, University of California, Merced, CA USA; 3 Faculty of Sport and Health Sciences, University of Jyväskylä, Jyväskylä, Finland; 4 Erasmus School of Economics, Erasmus University Rotterdam, Rotterdam, The Netherlands; 5 Tinbergen Institute, Erasmus University Rotterdam, Rotterdam, The Netherlands; 6 Tinbergen Institute and Erasmus Research Institute of Management, Erasmus University Rotterdam, Rotterdam, The Netherlands; 7 Department of Internal Medicine, Erasmus MC, Rotterdam, The Netherlands

**Keywords:** Preeclampsia, Cardiovascular health, Physical activity, Perceived determinants, Qualitative study

## Abstract

**Background:**

The objective of this study was to (1) qualitatively identify the perceived determinants of physical activity among women who have experienced severe preeclampsia, and (2) examine whether these determinants are consistent with the overarching processes outlined in the integrated behavior change (IBC) model, a novel model that describes physical activity as being a result of motivational, volitional, and automatic processes.

**Methods:**

Patients (*n* = 35) of the Follow-Up PreEClampsia (FUPEC) Outpatient Clinic, Erasmus MC, the Netherlands, participated in an anonymous online survey. The main outcomes under study were their perceived determinants of physical activity. Responses were analyzed using thematic analysis.

**Results:**

Thirteen themes emerged from the analysis. Six themes corresponded with motivational processes (future health, perceived ability, attitude, future reward or regret, physical appearance, and doing it for others), two with volitional processes (scheduling and planning), and two with automatic processes (affect and stress). Three themes were classified as environmental factors (time constraint, social support, and physical environment).

**Conclusions:**

A range of facilitating and hindering factors were described by women with prior severe preeclampsia as the determinants of their physical activity. These factors corresponded well with the overarching motivational, volitional, and automatic processes described in the IBC model. In addition, motivational and environmental factors beyond the IBC model were described. Addressing these perceived determinants could enhance the efficacy of physical activity interventions in this population.

*Tweetable abstract:* Motivational, volitional, automatic, and environmental factors drive physical activity in women with prior severe preeclampsia.

**Supplementary Information:**

The online version contains supplementary material available at 10.1186/s12905-022-01692-3.

## Introduction

Preeclampsia has been associated with a two- to eightfold increase in lifetime risk for cardiovascular disease (CVD) [1–3]. Therefore, cardiovascular follow-up and risk management are recommended for women with prior preeclampsia [2, 3]. CVD risk can be substantially reduced by engaging in sufficient levels of moderate-to-vigorous physical activity (MVPA) [4]. International guidelines advise adults to accumulate at least 150 min of moderate physical activity, or 75 min of vigorous physical activity, or an equivalent combination of MVPA spread throughout the week [4]. Over 31% of women worldwide fail to meet these guidelines [5]. Consequently, efficacious MVPA interventions are warranted, especially for priority groups such as women with prior severe preeclampsia [6].

Interventions promoting MVPA seldom achieve large and long-term effects [7]. A primary reason for these limitations could be their insufficient foundations in behavioral theory [8–11]. It is increasingly recognized that to maximize their efficacy, behavioral interventions should be based on theories that account for multiple processes that drive behavior [12–14]. Dual-system theories describe two types of processes that lead to action: *automatic* processes, determining behavior by impulses and habitual associations between context and action, and *deliberative* processes, determining behavior by reasoned deliberation and the value attached to the action [14–17].

To account for these multiple processes and provide more comprehensive explanations of behavior, integrated theories that derive their hypotheses from more than one theory have been proposed. A novel theory in this regard is the integrated behavior change (IBC) model. The IBC model integrates several well-established behavioral theories and posits that three types of processes determine MVPA: motivational, volitional, and automatic processes [18–20]. Motivational processes are modelled by variables that represent deliberative decision making, such as intention and intrinsic motivation. To follow, the IBC model proposes that the enactment of intentions formulated in the motivational phase are facilitated in the volitional phase by planning variables. Finally, automatic processes are represented by variables that bypass the intention-mediated processes, such as affect and habit. Since its conception, the IBC model has been used to explain a number of health behaviors, including MVPA, in observational studies [21–29].

Both quantitative and qualitative methods have been previously used to assess the *perceived* determinants of MVPA, and to offer recommendations for the design of MVPA interventions in the general postpartum population [30–32], and in women with prior preeclampsia specifically [33, 34]. These studies provide broad, rich data on the perceived determinants of MVPA, thereby contributing converging evidence of theoretical frameworks. Previously, only one comparable qualitative study used a theoretical framework, the theory of planned behavior, to interpret their results [35]. The IBC model has been qualitatively assessed only once before, in the context of MVPA in older adults [36].

The objective of this study is twofold. First, we aim to qualitatively identify the perceived determinants of MVPA among women who have experienced severe preeclampsia. Second, we aim to examine whether the identified determinants are in line with the overarching motivational, volitional, and automatic processes described in the IBC model. Both contributions may have utility in the development of effective MVPA interventions in women with prior preeclampsia.

## Method

The study follows the Standards for Reporting Qualitative Research (SRQR) guidelines [37].

### Study setting

The current study was conducted in the context of an outpatient clinic for women with prior severe preeclampsia. In the Erasmus Medical Center (Erasmus MC), cardiovascular follow-up and care is provided to women with prior severe preeclampsia at the multidisciplinary Follow-Up Pre-EClampsia Outpatient Clinic (FUPEC), the only clinic of its kind in the country [38]. There are currently around 1500 patients registered at the clinic, with an additional 100 to 150 women enrolling each year.

### Study population

Participants were recruited at the FUPEC clinic between September and November 2020 (*n* = 35). Inclusion criterion for participation was having experienced at least one pregnancy complicated by severe preeclampsia, as defined by the American Congress of Obstetricians and Gynecologists [39]. Exclusion criteria for participation were: < 18 years of age, pregnant at time of inclusion, < 3 months after delivery, any circumstance preventing MVPA (e.g. illness, injury, surgery, rehabilitation), insufficient knowledge of the Dutch language, and no possession of a smartphone. These exclusion criteria were applied to obtain a sample of women similar to those who will participate in a future app-based cardiovascular health promotion intervention [40]. A total of six women were excluded (three women were < 3 months after delivery; three women had insufficient knowledge of Dutch). Invited women were informed that participation in the study was voluntary and that they could withdraw from the study at any point without having to provide a reason. Women who chose to participate signed an informed consent form in advance of participation. The inclusion of participants was halted when the first two authors (LLK, MvdB) agreed that no new themes were expected to emerge from the inclusion of subsequent participants [41].

### Patient and public involvement

Patients and members of the public were not involved in the design, conduct, or reporting of this study.

### Design

An anonymous online survey was administered.

### Sampling strategy

The study used criterion sampling, i.e. participants needed to have prior experience with severe preeclampsia [42].

### Implementation

Women were asked at their FUPEC appointment whether they were interested in participating in an anonymous online survey. Women who did not make it to their scheduled appointment were asked by email. Those that indicated interest either at the appointment or by email received the survey. Of the 122 women approached, 119 agreed to receive the survey. Of those 119 women, 55 started the survey. Of those 55 women, 35 provided complete responses. Only complete responses were used in the current analysis. Women who did not provide complete responses (*n* = 20) were comparable to the study sample (*n* = 35) in age, educational level, when they had experienced severe preeclampsia, and whether or not, on an average week, they reached 150 min of MVPA (data not shown). The survey assessed four topics: demographics, needs for a cardiovascular health promotion intervention, perceived determinants of MVPA, and preferences for an a cardiovascular health promotion intervention. The current study used data on the first and third topics. Data on the second and fourth topics was collected for the purpose of a needs and preferences assessment, the results of which will be published separately. The survey was hosted online on the data capture tool Limesurvey [43]. Data were imported into IBM SPSS Statistics and NVivo for analyses [44, 45].

### Main outcome measures

The main outcome of this study was participants’ perceived determinants of MVPA, measured by five open questions in the anonymous online survey. These questions were based on prior qualitative research on MVPA [30–32, 34, 36, 46]. Women answered the questions by typing their answers in open text fields.

In order to tap into the motivational and volitional processes influencing MVPA, participants reported their general and preeclampsia-specific facilitators of and barriers to MVPA by answering the following questions: “What are your reasons for being physically active?”, “What makes it easier for you to be physically active?”, and “What makes it harder for you to be physically active?”. In addition, in order to tap into the automatic processes influencing MVPA, participants were prompted to recall a specific time when they had engaged in MVPA in the past (“Think of a time you have been physically active”), and to report on thoughts and feelings prior to engaging in MVPA by answering the following questions: “What thoughts went through your head in that moment?”, and “What emotions did you feel in that moment?”.

Participants also reported their demographic characteristics: age (years), number of children (number), living situation (with or without partner, with or without children); educational level (lower, middle, higher; classified using the International Standard Classification of Education [47]); paid employment status (yes, no; if yes, number of hours per week); when they had experienced severe preeclampsia (between three months and one year ago, between one and three years ago, over three years ago); whether their preeclampsia-related complaints were still present (yes, no; if yes, what complaints); whether or not, on an average week, they reached 150 min of MVPA (yes, no); and whether COVID-19 restrictions had an effect on their MVPA (yes, no).

### Data analysis

**Fig. 1:**
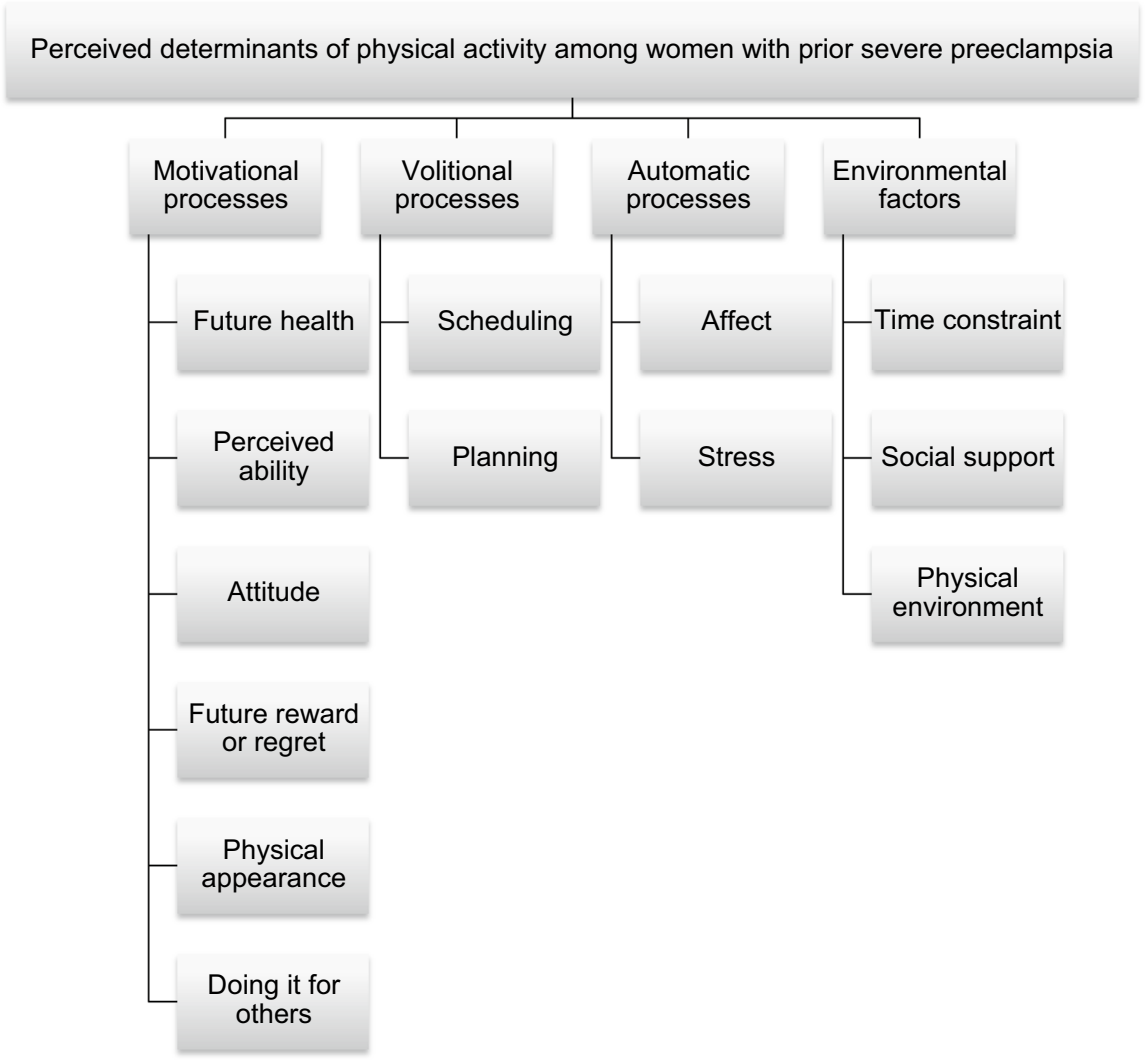
The coding tree of the thematic analysis

Descriptive statistics were used to report participants’ demographic characteristics. Thematic analysis was used to identify themes across the data [48–50]. After reading and re-reading participants’ responses, LLK and MvdB defined coding instances, and identified thirteen recurring themes in these instances. They then returned to the data independently, and categorized each coding instance into one of the thirteen themes. Initial interrater percent agreement was 71%. Subsequently, categorizations were jointly revisited until 100% agreement was reached. Finally, identified themes were matched to the overarching motivational, volitional, and automatic processes described in the IBC model, resulting in the coding tree of the current data (see Fig. 1) [20].

## Results

### Characteristics of the study sample

Table 1 shows the characteristics of the study sample (*n* = 35). Participants had a median age of 35 years. Most women had one child (54%), and were living with a partner (80%). The majority were highly educated (80%), and in paid employment (80%). Those in paid employment worked a median of 28 h per week. Most women experienced severe preeclampsia more than three years ago (54%). Almost half of women were still experiencing health complaints related to severe preeclampsia (49%), e.g., fatigue and anxiety, and problems with concentration and memory (examples of participants’ complaints are published under Additional file 1: Table S1). One in two women reported that they did not reach 150 min of MVPA per week (51%). The majority of women reported that the COVID-19 pandemic did not affect their MVPA levels (60%).

**Table 1:** Characteristics of the study population

Demographics		Total (*n* = 35)
Age*	Years	35 [32, 44]
Number of children	0	2 (6%)
	1	19 (54%)
	2	12 (34%)
	3	2 (6%)
Living situation	With partner and children	26 (74%)
	Without partner, with children	7 (20%)
	With partner, without children	2 (6%)
	Without partner and children	0 (0%)
Educational level**	Lower	0 (0%)
	Middle	7 (20%)
	Higher	28 (80%)
Paid employment	Yes	28 (80%)
	No	7 (20%)
	If yes, hours/week*	28 [20, 32]
*Preeclampsia characteristics*		
Time since severe preeclampsia	≥ 3 months to 1 year	8 (23%)
	1–3 years	8 (23%)
	≥ 3 years	19 (54%)
Preeclampsia related health complaints still present	Yes	17 (49%)
	No	18 (51%)
*Physical activity*		
Reaching ≥ 150 min of MVPA*** per week	Yes	17 (49%)
	No	18 (51%)
COVID-19 effects on MVPA	No effect	21 (60%)
	Negative	12 (34%)
	Positive	2 (6%)

Displayed value is frequency (percentage of total participants) unless marked with a *, in which case the displayed value is the median [interquartile range]

**Classified using the International Standard Classification of Education

***MVPA moderate-to-vigorous physical activity

### Overview of overarching themes

Figure 1 shows the coding tree of the thematic analysis. In total, thirteen themes emerged from the analysis. These themes were matched to four overarching themes: motivational processes [a–f], volitional processes [g–i], automatic processes [j–l], and environmental factors [m–o].

### Overview of themes

Themes within overarching themes are presented in descending order. Six themes corresponded with motivational processes: future health [a], perceived ability [b], attitude [c], future reward or regret [d], physical appearance [e], and doing it for others [f]. Two themes corresponded with volitional processes: scheduling [g], and planning [h]. Two themes corresponded with automatic processes: affect [i], and stress [j]. Finally, three themes were classified as environmental factors: time constraint [k], social support [l], and physical environment [m]. Example quotes of each theme are presented below in English (example quotes in their original language can be found under Additional file 1: Tables S2–S5). The prevalence of themes in the total number of participants is displayed in Table 2, and in the total number of quotes in Table 3.

**Table 2:** Prevalence of themes in total number of participants

Overarching themes and themes	Number of participants mentioning theme (Percentage of total participants *n* = 35)
*Motivational processes*	
a. Future health	35 (100%)
b. Perceived ability	26 (66%)
c. Attitude	17 (49%)
d. Future reward or regret	14 (40%)
e. Physical appearance	10 (29%)
f. Doing it for others	6 (17%)
*Volitional processes*	
g. Scheduling	10 (34%)
h. Planning	5 (14%)
*Automatic processes*	
i. Affect	26 (77%)
j. Stress	7 (20%)
*Environmental factors*	
k. Time constraint	23 (66%)
l. Social support	22 (63%)
m. Physical environment	20 (57%)

**Table 3:** Prevalence of themes in total number coding instances

Overarching themes and themes	Number of coding instances mentioning theme (Percentage of total coding instances *n* = 411)
*Motivational processes*	
a. Future health	97 (24%)
b. Perceived ability	54 (13%)
c. Attitude	33 (8%)
d. Future reward or regret	18 (4%)
e. Physical appearance	10 (2%)
f. Doing it for others	7 (2%)
*Volitional processes*	
g. Scheduling	14 (3%)
h. Planning	6 (1%)
*Automatic processes*	
i. Affect	48 (12%)
j. Stress	8 (2%)
*Environmental factors*	
k. Time constraint	45 (11%)
l. Social support	32 (8%)
m. Physical environment	36 (9%)

### Motivational processes

#### Future health

All women (100%) mentioned their future health, including physical, mental, and general health, as a motivator of their MVPA. Women reported their physical health as a motivator to be physically active, for example “Preparation for a healthy next pregnancy”, and “If I stay healthy and maintain my weight, I will have a lower chance for cardiovascular diseases”. Participants also mentioned their mental health as a facilitator of their MVPA, for example “Feeling good mentally”, and “I recover better mentally if I feel well physically”. Women also reported general health, i.e. health states that reflect both mental and physical well-being, as a motivator of their MVPA, for example “I want to feel less tired”, and “I want to overcome my constant fatigue”.

#### Perceived ability

The majority of women (66%) mentioned their perception of (temporarily) having a reduced ability to engage in MVPA as a barrier to their MVPA, for example “Many episodes of headaches”, and “Afraid to be intensely physically active after preeclampsia”.

#### Attitude

Approximately half of the women (49%) reported on their attitude towards physical activity. Most have reported their attitude to facilitate their MVPA, for example “I don’t see exercising as an obligation, but as something pleasant”, while a few reported it as a barrier, for example “I’d rather do other things”.

#### Future reward or regret

More than one-third of women (40%) mentioned an expected future reward or regret to motivate their MVPA, for example “As soon as you finish you will feel great”, and “I will have a bad conscience if I don’t go”.

#### Physical appearance

Approximately one-third of participants (29%) reported physical appearance as a facilitator of their MVPA, for example “To keep my body beautiful”.

#### Doing it for others

Almost one-fifth of women (17%) mentioned other people as a motivator of their MVPA, for example “I want to set a good example for my daughter”.

### Volitional processes

#### Scheduling

Over one-third of women (34%) reported scheduling to promote their MVPA, for example “Friends that I made an arrangement to exercise with”, and “Obligation [to attend physiotherapy]”.

#### Planning

Some women (14%) mentioned adequate planning as a facilitator of their MVPA, for example “A good planning””.

### Automatic processes

#### Affect

The majority of women (77%) reported specific feelings prior to participating in MVPA, for example “Fear”, and “Happy”.

#### Stress

One-fifth of women (20%) mentioned stress in their daily life as an obstacle to their MVPA, for example “Little relaxation”, and “Being overstimulated after a long day”.

### Environmental factors

#### Time constraint

Two-thirds of women (66%) reported time constraint as a barrier to their MVPA, for example “I need to have enough time to exercise, so that I don’t feel hastened to finish too soon”, and “Much to do at home with the baby”.

#### Social support

Almost two-third of participants (63%) discussed social support as a facilitator of their MVPA, for example “Friends to walk with”, and “Encouragement from FUPEC [doctors]”.

#### Physical environment

Over half of women (57%) reported their physical environment as a determinant of their MVPA, for example “Bad weather”, and “Sports facilities I really like”.

## Discussion

The objective of this qualitative study was to identify the perceived determinants of MVPA among women who have experienced severe preeclampsia, and to examine the extent to which these determinants relate to those proposed in the IBC model. Our findings demonstrate that women with prior severe preeclampsia perceive a wide range of facilitating and hindering factors to determine their MVPA. In total, thirteen themes emerged from the analysis. These themes were matched to four overarching themes: motivational processes, volitional processes, automatic processes, and environmental factors. We found these themes to correspond well with the overarching processes identified in the IBC model. In addition, motivational and environmental factors beyond the IBC model were reported by participants.

### Interpretation of key findings

Our study provides detailed data on the perceived determinants of MVPA in women with prior severe preeclampsia. All women reported concerns about their future health, such as reducing their future risk for CVD, as a motivator of their MVPA. While future health has been previously found to be a motivator for adopting a healthy lifestyle in women with prior preeclampsia [35], the prevalence of this facilitator in our population is noteworthy, considering that past studies found approximately one-tenth of postpartum women to report their future health as an important motivator of their MVPA [31]. Two-third of our participants reported their perceived ability to engage in MVPA to be reduced temporarily by for example headaches: about three times more often than the general postpartum population [31]. A link between preeclampsia and migraine headaches has previously been hypothesized [51]. Our finding that fatigue and stress are perceived by many as a barrier to MVPA is consistent with prior findings in postpartum women [30]. Some women in our study were hoping to alleviate their health complaints by engaging in MVPA, a mechanism backed by research [52–54], and a wish echoed by other postpartum women [31]. Some women aimed to accommodate healthy future pregnancies by engaging in MVPA, in line with previous research that found preparation for a future pregnancy, and the young family stage in general, to provide unique motivation to engage in MVPA [32, 55–57].

In general, we found that the perceived determinants of MVPA among our participants had reasonable correspondence with the overarching motivational, volitional, and automatic processes described in the IBC model [20]. In addition, our study identified a motivational determinant of MVPA beyond those described in the IBC model: future reward or regret. Future reward and future regret have been previously identified as potential determinants of MVPA in the general population [58–60]. Furthermore, in addition to the processes described in the IBC model, we found the perceived environmental factors of time constraint, social support, and physical environment to influence MVPA, in line with prior research in postpartum women [30–32]. Basing behavioral interventions on theory, and qualitatively assessing the choice of theory during the design stage, can add to the efficacy of subsequent interventions [61]. In combination with evidence from previous observational studies [21–29], the results of our study suggest that the IBC model, potentially extended with the variables of future reward or regret, time constraint, social support, and physical environment, may be a suitable theory-base for MVPA interventions in women with prior severe preeclampsia.

### Implications for practice

Results from this study provide entry points for improving lifestyle counseling at the clinic, and for other types of lifestyle interventions that health care practitioners may use to promote MVPA in women with prior preeclampsia. Most participants expressed their appreciation for existing support, and requested additional support from their healthcare professionals in their quest for sufficient MVPA, in line with prior research in this population [33, 34].

In light of our finding that many women perceive themselves to be less able to engage in MVPA after having experienced severe preeclampsia, practitioners are encouraged to convey to their patients that clinical guidelines advise them to accumulate as much MVPA as the general population [2]. Given evidence on the reciprocal relationship between mental health and MVPA [54], and on the negative impact of preeclampsia on mental health [62, 63], practitioners could emphasize to their patients that engaging in MVPA will not only benefit their physical health, but also their mental health.

Several volitional processes described by our participants resembled some well-established behavior change techniques, i.e. the active ingredients of behavior change interventions [64]. The scheduling described by our participants can be likened to temptation bundling, i.e. linking an action one wants to do with an action one needs to do [65], and commitment, i.e. pre-committing oneself to MVPA by ways of financial or social investment [66]. Some participants used planning to stay active [67]. Given these techniques’ apparent relevance, and similarity to techniques previously suggested to aid the MVPA of postpartum women [30], they could be valuable components of MVPA interventions in this population.

### Strengths and limitations

Our study has several strengths. Our findings provide health care practitioners with insight into the perceived determinants of MVPA in women with prior severe preeclampsia, allowing them to tailor lifestyle counselling to their patients’ needs. Second, our comparison of the perceived determinants of MVPA with a novel theoretical framework, the IBC model, supports health care practitioners in providing care in a theory-based manner. Finally, our findings may be applicable to other populations, such as women with other types of prior hypertensive pregnancy disorders, or other pregnancy complications, such as gestational diabetes or intrauterine growth restriction. However, some limitations should be considered when interpreting the results of our study. It is possible that our study population, self-selected from an outpatient clinic specialized in the cardiovascular follow-up and risk management of women with prior severe preeclampsia, may have had a higher awareness of their increased risk for CVDs than the average woman with prior preeclampsia. Second, as most of our participants were highly educated, our findings are less generalizable to all socioeconomic groups. Third, some quotes of participants provided little context, which limited the interpretation of the reported determinant (e.g. whether it is a facilitator or barrier of MVPA). Fourth, the relationship between perceived environmental factors and *actual* environmental factors was not assessed in this study; therefore, it is possible that the perceived environmental factors reported by participants more closely reflect (a lack of) perceived behavioral control, or other individual-level variables, rather than true environmental constraints. Finally, participating in a qualitative study requires deliberative thought, which means that our data collected on the automatic determinants of MVPA reflect individuals’ perceptions and experiences; whether or not participants are aware of, or have access to, processes that are automatic and are purported to affect behavior beyond their awareness is an open question [68].

## Conclusion

A wide range of factors determine MVPA among women with a history of severe preeclampsia. The identified factors correspond well with the overarching motivational, volitional, and automatic processes described in the IBC model. In addition, motivational and environmental factors beyond the IBC model were identified. Targeting these factors could enhance MVPA intervention efficacy among women with prior severe preeclampsia.

## Supplementary Information

**Additional file 1.** Supporting information.

## Data Availability

Examples of participant quotes are published in this article under supporting information. Participants were not asked to provide informed consent for the sharing of their data with third parties, therefore, data will not be made available publicly or to other researchers.
