# Supplementary material for: Perceived determinants of physical activity among women with prior severe preeclampsia: a qualitative assessment
Source: BMC Womens Health. 2022 Apr 27;22:133. doi: 10.1186/s12905-022-01692-3 (PMC9043879; doi:10.1186/s12905-022-01692-3)
Supplement: Supplementary file 1 — Additional file 1. Supporting information. [file 12905_2022_1692_MOESM1_ESM.docx]

**Supporting information**

**Table 1. Examples of participant quotes: health complaints still present due to prior severe preeclampsia**

| **Quote in Dutch**  **(Original)** | **Quote in English**  **(Translation)** |
| --- | --- |
| “Hoofdpijn, prikkelgevoelig, angstig, soms moe” | “Headache, sensitive to stimuli, anxious, sometimes tired” |
| “Geheugen-, concentratie-, leer-, oriëntatieproblemen, zeer prikkelgevoelig, zeer vermoeid” | “Problems with memory, concentration, learning and orientation, very sensitive to stimuli, very tired” |
| “Hypertensie” | “Hypertension” |
| “Korte termijn geheugen” | “Short-term memory” |
| “Ik ben nog snel moe en heb minder energie” | “I am still quickly fatigued and have less energy” |
| “Snel overprikkeld, niet op woorden kunnen komen, mindere concentratie” | “Easily overstimulated, unable to find words, less concentration” |
| “Mijn mentale draagkracht en conditie zijn nog lager” | “My mental capacity and condition are still lower [than before preeclampsia]” |
| “Ik heb nog steeds een hoge bloeddruk” | “I still have high blood pressure” |
| “Vergeetachtig, traumatische beelden zien, warrig” | “Forgetful, seeing traumatic images, confused” |

**Table 2. Examples of participant quotes: Motivational processes**

| **Theme** | **Quote in Dutch**  **(Original)** | **Quote in English**  **(Translation)** |
| --- | --- | --- |
| 1. Future health | “Conditie opbouwen” | “To build up my physical stamina” |
| 1. Future health | “Afvallen” | “Lose weight” |
| 1. Future health | “Als ik gezond en op gewicht blijf heb ik minder kans om hart- en vaatziekten te krijgen” | “If I stay healthy and maintain my weight, I will have a lower chance for cardiovascular diseases” |
| 1. Future health | “Voorbereiding op een gezonde volgende zwangerschap” | “Preparation for a healthy next pregnancy” |
| 1. Future health | “Extra afvallen omdat de zwangerschapskilo's er toch minder snel afgaan na zo'n heftige tijd.” | “I want to lose the baby weight, it disappears slower after such challenging times” |
| 1. Future health | “(Om voldoende te bewegen) om zo gezond mogelijk te leven” | “I am physically active to stay as healthy as possible” |
| 1. Future health | “Minder moe willen zijn” | “I want to feel less tired” |
| 1. Future health | “Goed voor mijn herstel en gezondheid” | “Good for my recovery and health” |
| 1. Future health | “De constante vermoeidheid willen overwinnen” | “I want to overcome my constant fatigue” |
| 1. Future health | “Goed voelen mentaal” | “Feeling good mentally” |
| 1. Future health | “Ik herstel beter mentaal, als ik me fysiek ook goed voel” | “I recover better mentally if I feel well physically” |
| 1. Perceived ability | Vaak hoofdpijn | “Many episodes of headaches” |
| 1. Perceived ability | “Nog lichte pijn aan keizersnede” | “Still having light pain because of my caesarean” |
| b. Perceived ability | “Vermoeidheid door prikkelgevoeligheid | “Fatigue due to sensitivity to stimulation” |
| b. Perceived ability | “Angst om intensief te bewegen na pre-eclampsie” | “Afraid to be intensely physically active after preeclampsia” |
| c. Attitude | “Zie het niet als verplichting maar echt als iets leuks!” | “I don’t see exercising as an obligation, but as something pleasant!” |
| 1. Attitude | “Doe liever andere dingen” | “I’d rather do other things” |
| 1. Future reward or regret | “Zodra je klaar ben voel je jezelf super” | “As soon as you finish you will feel great” |
| d. Future reward or regret | “Straks voel ik mij fitter” | “I immediately feel more fit” |
| d. Future reward or regret | “Het gevoel van een slecht geweten” | “I will have a bad conscience if I don’t go” |
| 1. Physical appearance | “Om mijn lichaam mooi te houden” | “To keep my body beautiful” |
| 1. Doing it for others | “Goed voorbeeld voor dochter” | “I want to set a good example for my daughter” |
| f. Doing it for others | “Laten zien aan de buitenkant dat er niks mis was” | “I wanted to show the world that there is nothing wrong” |

**Table 3. Examples of participant quotes: Volitional processes**

| **Theme** | **Quote in Dutch**  **(Original)** | **Quote in English**  **(Translation)** |
| --- | --- | --- |
| g. Scheduling | “Sociale aspect” | “Social aspect” |
| 1. Scheduling | “Vrienden waarmee beweegafspraak is gemaakt” | “Friends that I made an arrangement to exercise with” |
| g. Scheduling | “Verplichting [van de fysiotherapie]” | “Obligation [to attend physiotherapy]” |
| 1. Planning | “Na een lange werkdag nog een spinnig les doen, gaat helaas niet. Wel een rustigere yogales bijvoorbeeld. De intense lessen plan ik op mijn vrije dagen”. | “After a long day at work I cannot do a spinning class, but I can do a yoga lesson. I plan to attend more vigorous lessons on free days” |
| 1. Planning | “Goed plannen” | “A good planning” |

**Table 4. Examples of participant quotes: Automatic processes**

| **Theme** | **Quote in Dutch**  **(Original)** | **Quote in English**  **(Translation)** |
| --- | --- | --- |
| 1. Affect | “Blij” | “Happy” |
| i. Affect | “Ben trots dat ik zo hard heb gewerkt” | “I am proud that I have worked this hard” |
| i. Affect | “Frustratie” | “Frustration” |
| i. Affect | “Angst” | “Fear” |
| 1. Stress | “Stress” | “Stress” |
| 1. Stress | “Weinig ontspanning” | “Little relaxation” |
| j. Stress | “Overprikkeld zijn na een lange dag” | ”Being overstimulated after a long day” |

**Table 5. Examples of participant quotes: Environmental factors**

| **Theme** | **Quote in Dutch**  **(Original)** | **Quote in English**  **(Translation)** |
| --- | --- | --- |
| k. Time constraint | “Of heb ik voldoende tijd om te sporten zodat ik tijdens het sporten me niet opgejaagd voel om het snel af te ronden.” | “I need to have enough time to exercise, so that I don’t feel hastened to finish too soon.” |
| k. Time constraint | “Veel te doen thuis met baby” | “Much to do at home with the baby” |
| l. Social support | “Vrienden om even te wandelen” | “Friends to walk with” |
| l. Social support | “Meer tips en vaste plan van en professional” | “More tips and a steady plan from a professional” |
| l. Social support | “Aansporing vanuit FUPEC” | “Encouragement from FUPEC” |
| m. Physical environment | “Buiten sporten” | “Exercising outside” |
| m. Physical environment | “Slecht weer” | “Bad weather” |
| m. Physical environment | “Flexibele tijden” | “Flexible times” |
| m. Physical environment | “Sportfaciliteiten die ik echt leuk vind” | “Sport facilities I really like” |
